# Mechanical Properties of Rice Husk Biochar Reinforced High Density Polyethylene Composites

**DOI:** 10.3390/polym10030286

**Published:** 2018-03-08

**Authors:** Qingfa Zhang, Weiming Yi, Zhihe Li, Lihong Wang, Hongzhen Cai

**Affiliations:** 1 School of Agricultural and Food Engineering, Shandong University of Technology, Zibo 255000, China; zhangqingfacll@126.com (Q.Z.); yiweiming@sdut.edu.cn (W.Y.); lizhihe@sdut.edu.cn (Z.L.); wanglh@sdut.edu.cn (L.W.); 2 Shandong Research Center of Engineering and Technology for Clean Energy, Zibo 255000, China

**Keywords:** rice husk biochar, HDPE, composites, DSC, mechanical properties

## Abstract

Rice husk biochar was utilized to reinforce high-density polyethylene (HDPE) and to prepare biochar/plastic composites (BPC) by the extrusion method. Morphologies, non-isothermal crystallization behavior, and mechanical properties of the composites were investigated. The SEM (scanning electron microscope) showed that HDPE was embedded into the holes of the rice husk biochar. The DSC (differential scanning calorimeter) showed that biochar could reduce the crystallization rate and the higher the content of rice husk biochar, the slower the crystallization rate. Significantly, the bending and tensile strength of BPC could reach 53.7 and 20 MPa, far beyond WPC (wood plastic composites). With the increase of filler content, BPC were still stronger than WPC, although the impact strength of BPC and WPC all showed a general decline in the trend. The strong interaction was achieved by the utilization of rice husk biochar to reinforce HDPE.

## 1. Introduction

Synthetic polymers have been widely used in WPC due to their chemically inactive, good abrasion resistance, and good deformability as matrix; wood fibers are increasingly used for reinforcement in thermoplastics due to their low density, good mechanical properties, unlimited availability, and low price [1], which is why WPC has made great progress over the past two decades. As a new type of biomaterials, WPC are normally produced by mixing wood fiber with plastic or by adding wood fiber in a polymer matrix and pressing or molding under high pressure and temperature [2]. Although WPC have received considerable attention relying on properties such as low friction coefficient, low abrasion, good plasticity, good burning resistance, and environmental performance, the mechanical properties are not good [3]. Many scientists have conducted a great deal of research about it and some progress has been made. The surface of the fiber contains a large number of polar hydroxyl groups, and the compatibility with non-polar or weak polar polymer materials is very poor. Therefore, it is necessary to modify the fiber to reduce polar hydroxyl groups. Heat treatment has a great influence on the strength of the fiber. Nguila et al. found that heat treatment could reduce the reactivity of wood flour, which was mainly because the degradation of the semi-fiber resulted in the decrease of the high activity hydroxyl groups during the heat treatment [4]. Acid and alkali can dissolve some pectin, lignin, and hemicellulose in the fiber, but not change the chemical structure of the main cellulose. The treatment of fiber with acid or alkali can increase the contact area between fiber and polymer and improve the mechanical properties of the materials [5,6]. In addition, the coupling agent could improve the compatibility of WPC. Many research findings revealed that maleic anhydride-grafted polyolefin can increase the mechanical strength of WPC and the dispersion of the fiber [7,8]. Lately, biochar has been added as an additive to improve the mechanical properties of WPC and proved feasible [9].

As a renewable material, biochar is produced through pyrolysis from a variety of agricultural and forestry wastes [10,11] and biochar has received attention from academia and industry as an effective soil amendment and remediation agent for organic contaminants [12,13]. Recently, biochar has also been used to prepare composites to improve mechanical strength due to its special structure. Many researchers have prepared composites with biochar as filler instead of fiber, the result showed that biochar is beneficial to improve the mechanical properties and flame retardation of the composites [14,15,16,17,18]. As a kind of the biochar, charcoal powder can not only be used as a filling material, but can also greatly improve the processing properties of ultrahigh molecular weight polyethylene (UHMWPE) [19]. Biochar/plastic composites (BPC) are environmentally attractive since biochar is renewable and the recycled thermoplastic plastics are available, it could be prepared by melt processing because of its high thermal stability and strong plasticity, just like WPC [20]. Additionally, some properties of WPC depend heavily on the crystallization behavior. Therefore, it is so valuable and important to study the crystallization behavior of the materials in order to control and optimize the properties. Considering the fact that BPC is under non-isothermal conditions, it has necessary and practical meaning to study the non-isothermal crystallization and its kinetics. 

In this paper, the extrusion production process was used to prepare BPC with HDPE and rice husk biochar obtained by rice husk pyrolysis. The crystallization behavior of BPC at different cooling rates was studied and Mo’s equation [21] was used to analyze the non-isothermal crystallization process and to study the characteristics of non-isothermal crystallization of BPC. The mechanical properties of BPC were presented and discussed to provide a theoretical basis for application.

## 2. Experiment

### 2.1. Materials

HDPE used was purchased from Qilu Petrochemical Co., Ltd. (Zibo, China) as the matrix material. TPW604 (Tianhe, China) was used as lubricant to reduce the friction between equipment and materials. The rice husk biochar was obtained by fast pyrolysis of rice husk powder at 500 °C using a fluidized bed reactor in a N_2_ atmosphere. The rice husk biochar and rice husk powder were sieved in order to keep them at less than 100 μm and dried in an oven at 105 °C for 24 h to reduce the moisture content to less than 2% prior to processing.

### 2.2. Preparation of Composites

To study the effect of the content of the rice husk biochar, 5 concentrations were chosen: 30, 40, 50, 60, and 70 wt %. The raw materials were dry blended by a high-speed mixer (JHN-15, Zhengzhou, China) for 10 min to obtain a homogeneous blend. In order to improve the experimental efficiency, the mixed materials were processed with twin screw extruder (BP-8177, Dongguan Baopin International Precision Instrument Co., Ltd., Guangzhou, China), in a temperature zone setting of 135, 145, 155, 165, 175, and 185 °C, the feeding and extrusion speed were 30 r/min. Finally, the samples were extruded from the mold. The samples were left for 24 h at room temperature for testing.

### 2.3. Measurements and Characterization

#### 2.3.1. Microscopy and Structure

The rice husk biochar and BPC were investigated with a field emission scanning electron microscope (FEI Sirion 200, Hongkong, China) operating at 20 kV. The powder and the fractured surface of impact section were sputtered with gold to avoid electrical charging during examination prior to processing.

#### 2.3.2. Non-Isothermal Crystallization Behavior

The pure HDPE and the five compounds were ground into powder for use and the non-isothermal crystallization were carried out using a differential scanning calorimeter analyzer (DSC-Q100, TA Instrument, Shanghai, China). The samples were rapidly heated at a rate of from 20 °C/min to 180 °C and held isothermally for 1 min to eliminate mechanical and thermal history, and then cooled to room temperature at 5, 10, 15, 20 °C/min, respectively, to record the crystallization enthalpies.

#### 2.3.3. Mechanical Properties

Bending, tensile, and impact strength were tested for the mechanical properties of different samples. The samples were cut into different sizes by the universal system prototype (ZHY-W, Chengde Testing Machine Co., Ltd., Chengde, China). The bending and the tensile strength were tested by an electronic universal testing machine (5969, Instron, Jinan, China), and their dimensions were 80 × 10 × 4 mm (GB/T 9341-2008, China) and 160 × 10 × 4 mm (GB/T 13525-92, China). The samples’ dimensions were 80 × 10 × 4 mm (GB/T 1843-2008, China), for the impact strength which was carried out with a pendulum electronic impact testing machine (JB-300B, Jinan Heng Think Grand Instrument Co., Ltd., Jinan, China). All mechanical tests of each composition were repeated at least five times, and the average values were adopted.

## 3. Results and Discussion

### 3.1. SEM Observations

**Figure 1:**
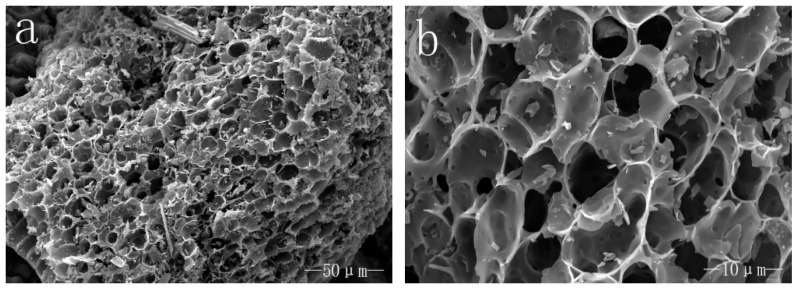
SEM images of the rice husk biochar: (**a**) 1000×; and (**b**) 5000×.

Figure 1a,b presents the typical morphology of the rice husk biochar at different magnifications. It shows that the internal structure of rice husk was destroyed due to high temperature action, but the rice husk biochar surface formed a pore structure with different pore size and the shape of the hole was round or oval. This is similar to the microstructure of carbonized wood [22,23] which was different from the study of Tzong-Horng Liou who thought that the outer epidermis of rice husk was well organized and had a corrugated structure [24]. The large differences in the microstructure between the rice husk and the rice husk biochar would lead to different properties when they are combined with the matrix as fillers, because a good deal of holes in the rice husk biochar might strengthen the interface with the polymer matrix [3,25].

**Figure 2:**
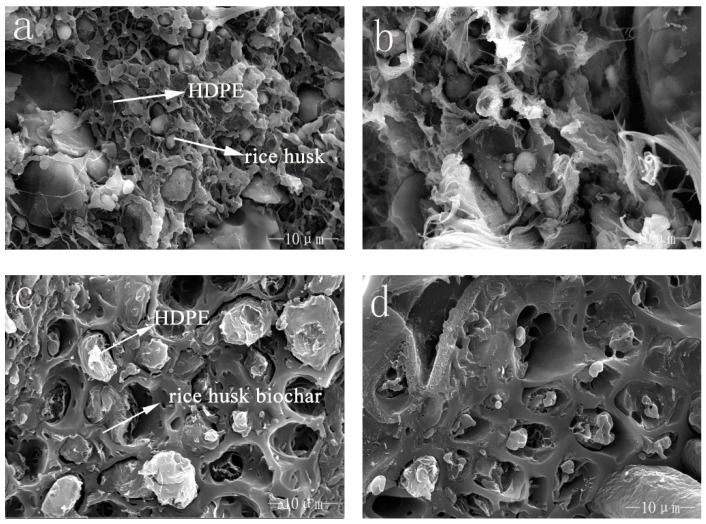
SEM images of the broken impact sections of WPC (**a**) rice husk—30 wt %; (**b**) rice husk—60 wt % and BPC (**c**) rice husk biochar—30 wt %; and (**d**) rice husk biochar—60 wt %.

Figure 2 shows the impact fracture surfaces of WPC and BPC under different magnification. Obviously, the combination of the two materials was significantly different. Figure 2a demonstrates that the rice husk powder can be fully covered by the HDPE matrix, so that it can be evenly distributed in the matrix to increase its contact area and the interface is good, which made the interface diffusion and mechanical interlocking a high degree. However, when the content of the rice husk powder is 60 wt %, the combination with HDPE became poor and the interface appeared obvious gaps. The interface of the composites was worse, the rice husk powder cannot be completely wrapped and part of the powder exposed to the outside from Figure 2b. This result is consistent with ordinary WPC [26]. Figure 2c shows that HDPE were embedded into the holes of the rice husk biochar. Additionally, biochar was held together by the viscosity of the HDPE so that the rice husk biochar and HDPE could combine tightly. From Figure 2d we can know that when the content of the rice husk biochar rose to 60 wt %, there still was a good interface quality appeared on the section rather than obvious gaps, but some rice husk biochar was not well dispersed in the matrix because of the lesser content of HDPE.

### 3.2. Non-Isothermal Crystallization Behavior

#### 3.2.1. Crystallization Behavior

**Figure 3:**
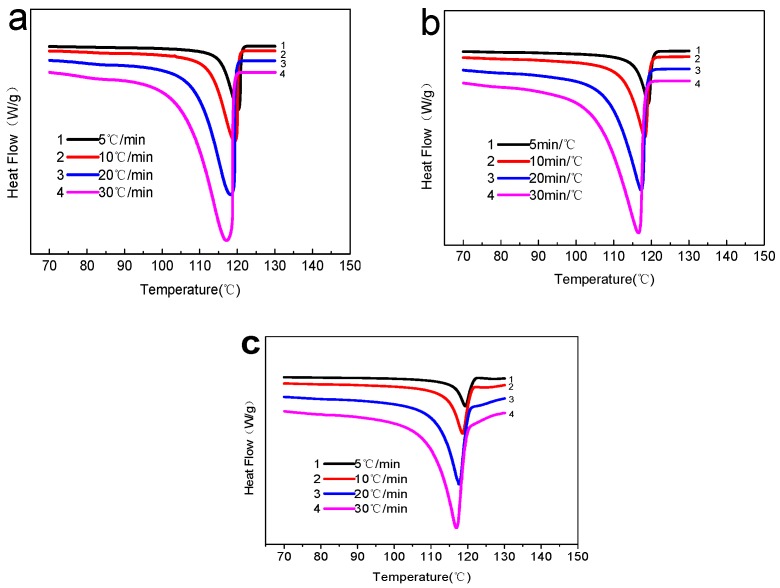
DSC curves of non-isothermal crystallization at different heating rates for different samples: (**a**) HDPE; (**b**) biochar—30 wt %; and (**c**) biochar—70 wt %.

Figure 3 shows the DSC curves of non-isothermal crystallization of HDPE and different BPC at different heating rates. The different materials under different cooling rates all could obtain obvious crystallization peaks. The analysis of the figures reveals that with the increase of cooling rate, the highest crystallization peak temperatures of pure HDPE [27] and BPC with different amounts of rice husk biochar moved toward lower temperature. The reason was that the increase of the cooling rate led to the increase of the degree of super-cooling in the crystallization, the temperature at which the crystallization started moved to the low temperature. At lower temperature, the activity of molecular chain was poor, and the degree of crystallization was also different, which was the reason why the crystallization peaks of HDPE became wider [28]. As for BPC, although the molecular activity decreased and the diffusion rate decreased under lower temperature, the nucleation rate increased because of large undercooling and more HDPE molecular chain can be discharged into the lattice easily. Thus, the degree of crystallization became worse, the range of crystallization temperature became larger, and the crystallization peaks became wider [29]. The maximum crystallization peak temperature of different samples at different cooling rate is reported in Table 1. It is obviously that the maximum crystallization peak temperature of all the samples decreased with the increase of cooling rate because of the increasing crystallization degree of super cooling and the content of biochar in HDPE caused a slightly decrease, which was different from WPC [30]. This may be ascribed to the weakened nucleating ability of biochar and improved interfacial adhesion between HDPE and biochar in the composites, where the movement of HDPE segments is inhibited. However, the content of biochar had little effect on the maximum crystallization peak temperature of the composites.

**Figure 4:**
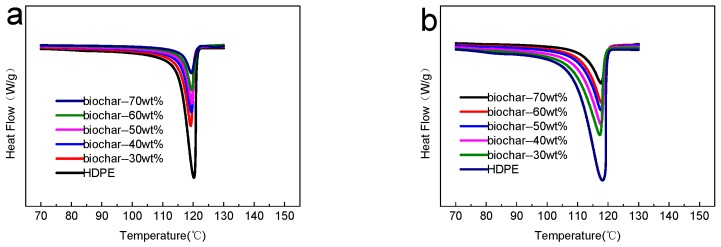
DSC curves of non-isothermal crystallization for different samples at different heating rates: (**a**) 5 °C/min; and (**b**) 20 °C/min.

Figure 4 presents the DSC curves of non-isothermal crystallization of HDPE and different composites at the heating rates of 5 and 20 °C/min. Figure 4 indicated that the content of biochar had no obvious effect on the maximum crystallization peak temperature, which was consistent with Table 1. Nevertheless, the content of biochar had a significant effect on the width of the peak. With the increase of the content of biochar, the crystallization peak became narrower under the same cooling rate, which stated clearly that the range of crystallization temperature of the composites became small. And this was because biochar hindered the molecular chain from moving toward the nucleus, which inhibited the crystallization of HDPE during the crystallization of HDPE [31].

#### 3.2.2. Non-Isothermal Crystallization Kinetics

A novel kinetic approach was proposed by Mo to describe the non-isothermal crystallization process, which combined the Avrami equation with the Ozawa equation. Mo’s equation [21] has been used for many times:

lnD=lnF(T)−alnt

where *F*(*T*) refers to the cooling rate which is required to achieve a certain degree of crystallinity in the unit crystallization time. *F*(*T*) is so important, because the smaller *F*(*T*), the higher the crystallization rate. *a* = *n*/*m* refers to the ratio of the Avrami constant to the Ozawa index and *t* refers to the crystallization time. 

**Figure 5:**
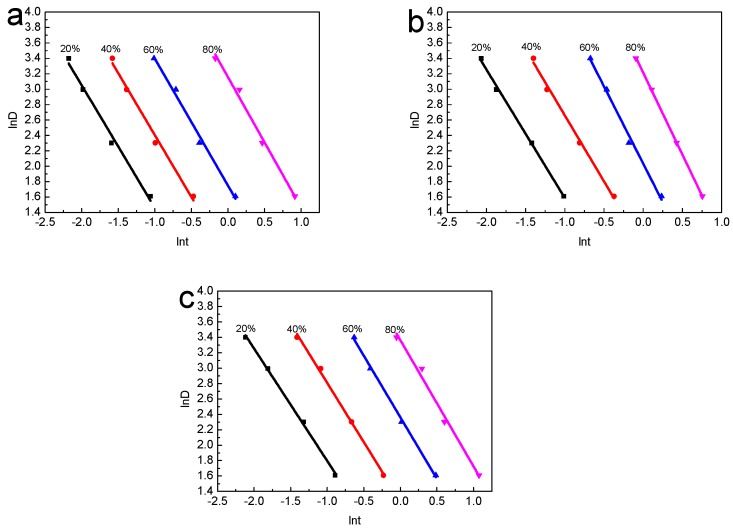
Plots of the ln*D* versus ln*t* for the non-isothermal crystallization of the samples: (**a**) HDPE; (**b**) biochar—30 wt %; and (**c**) biochar—70 wt %.

Figure 5 represents the curves of ln*D*~ln*t* for HDPE and different composites. A good linear relationship between ln*D* and ln*t* was shown in Figure 5 and it proved that it was feasible to study the non-isothermal crystallization kinetics of RHB/HDPE composite materials. The *F*(*T*) and *a* = *n*/*m* can be extracted from intercept and slope of the simulating line of Figure 5 and they were listed in Table 2.

Table 2 showed that the values of all the samples were almost the same, which was about 1.5. Additionally, the *F*(*T*) values increased with the increase of crystallinity for all the samples, which indicated that the cooling rate increased that required to achieve a certain degree of crystallization in the unit crystallization time. It can also be seen that the *F*(*T*) values of BPC were larger than that of pure HDPE at the same cooling rate, which showed that the cooling rate of HDPE was lower than that of BPC when the same crystallinity was reached. That meant that the crystallization rate of HDPE was greater than BPC, because rice husk biochar prevented the proliferation of molecular chains, and the crystallization rate slowed down. When the content of rice husk biochar reached 70 wt %, the *F*(*T*) values became bigger under different relative crystallinity, which meant that the higher the content of rice husk biochar, the slower the crystallization rate [28]. 

### 3.3. Mechanical Properties

#### 3.3.1. Bending Strength

**Figure 6:**
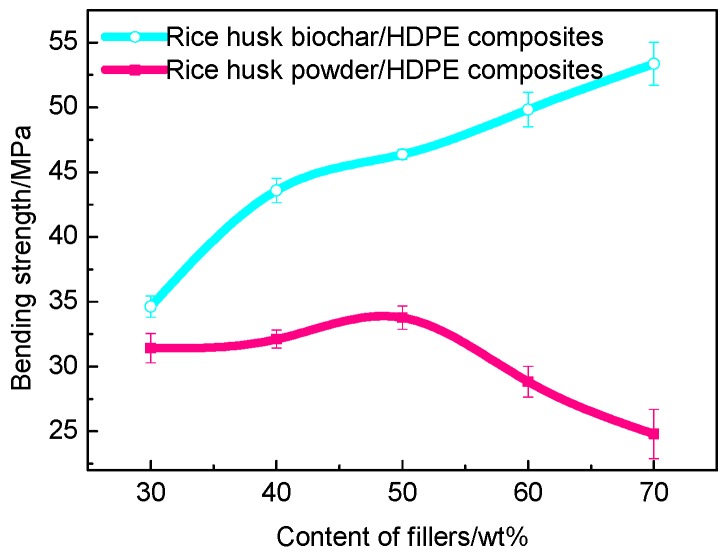
Bending strength of BPC and WPC.

The bending strength of composites with different fillers is presented in Figure 6. There was a very large difference between WPC and BPC in bending strength. The bending strength had been improved before the rice husk powder content increased to 50 wt % from Figure 6, which was because HDPE entered the gaps on the surface of the rice husk and wrapped it as an adhesive, and promoted the entanglement and contact between the rice husk powder. However, when the rice husk powder content is over 50 wt %, the bending strength began to decline and became lower and lower. The reason was: with the increase of the content of rice husk powder, the agglomeration in the HDPE matrix strengthened, which caused the stress concentration and defects in the composite materials [32]. Figure 6 also shows that the composites filled with the rice husk biochar showed higher bending strength than those of the composites filled with the rice husk powder. This was mainly due to the special combination which was completely different from WPC. Additionally, the strength increased as the content increased, the content of rice husk biochar was 70 wt % to reach the maximum 53.7 MPa. The reason of the result was that the rice husk biochar limited the movement of the HDPE chain and decreased the deformation capacity of the matrix in the elastic zone. 

#### 3.3.2. Tensile Strength

**Figure 7:**
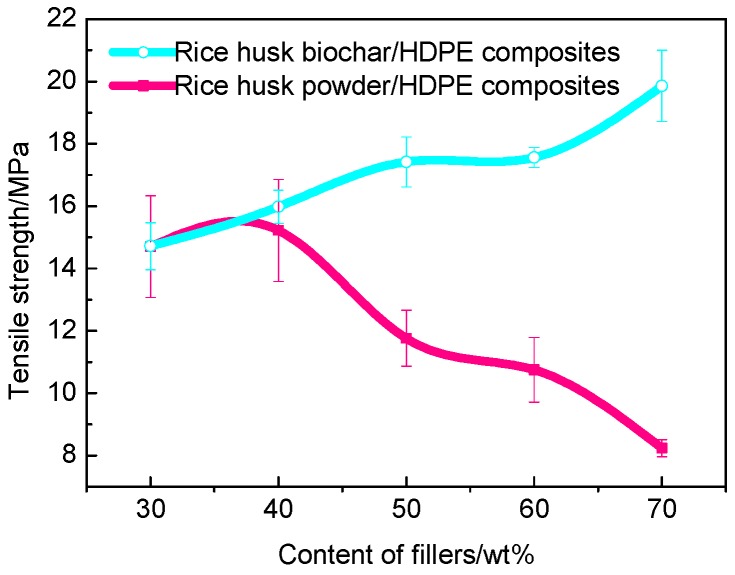
Tensile strength of BPC and WPC.

The tensile strength curves of composites containing different fillers are reported in Figure 7. The tensile strength of WPC is consistent with literature evidence [33]. From the curves, we can know that less rice husk powder can improve the tensile strength of composites, but with the further increase of rice husk content, there was a negative impact on its tensile strength. This is because less rice husk powder could be completely wrapped by HDPE, which made their contact more closely. On the contrary, less HDPE cannot wrap the rice husk powder completely, which weakened the interfacial adhesion between the rice husk powder and HDPE matrix [34]. Meanwhile, the tensile strength of BPC was shown compared to a typical WPC in Figure 7. After the addition of the rice husk biochar, the tensile strength was the same as WPC roughly. Whilst they differed in the tensile strength increased with the increase in the rice husk biochar content. The rice husk biochar was obtained by fast pyrolysis at 500 °C using a fluidized bed reactor, there was no hydrophilic hydroxyl functional group in the interior, which was beneficial to the interfacial bonding between biochar and HDPE. The rice husk biochar behaved as rigid particles in the polymer matrix, which restricted the movement of polymer chains. This once again proves that the rice husk biochar could reinforce HDPE compared with the rice husk powder.

#### 3.3.3. Impact Strength

**Figure 8:**
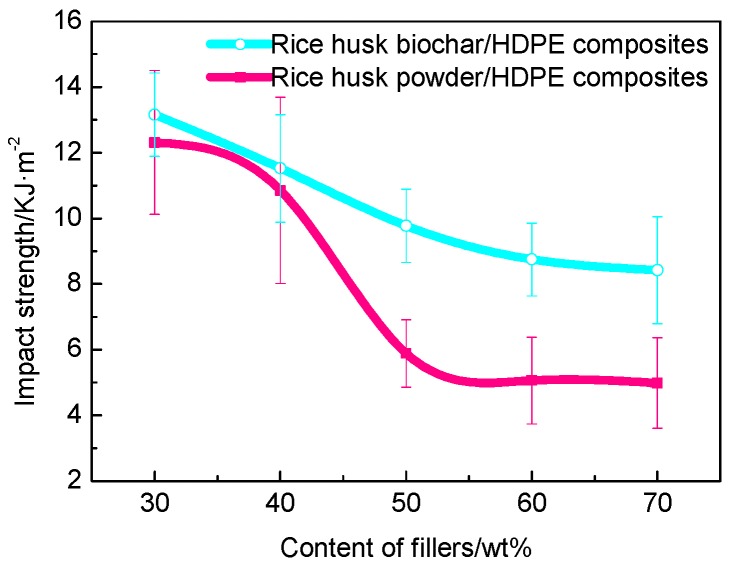
Impact strength of BPC and WPC.

The results of impact strength of composites are presented in Figure 8 showing that when the different fillers content is about 30 wt %, the impact strength of WPC and BPC is still relatively high which was because that fillers were well dispersed in the matrix, the strong interaction was achieved. With the increase of fillers, the impact strength of BPC and WPC all decreased. That is because with the increase of filler content, HDPE decreased and addition of fillers increased the rigidity of the composites while making the composites brittle and the toughness declining [35]. Even so, the impact strength of BPC was still higher than that of WPC, in general. The microscopy of BPC showed that the HDPE was embedded into the holes of the rice husk biochar instead of hooking up with each other which meant that the blend of biochar and HDPE was more uniform. Hence, BPC could absorb more impact energy than WPC.

## 4. Conclusions

The aim of this study was to delve into the effect of the rice husk biochar on the properties of BPC. It has been proved that the rice husk biochar can not only be used as a filling material, but also could improve the mechanical properties of composites. Scanning electron microscopy shows that HDPE was embedded into the holes of the rice husk biochar. DSC showed that the highest crystallization peak temperatures of pure HDPE and BPC with different amounts of rice husk biochar all moved toward low temperature, and the non-isothermal crystallization kinetics showed that the biochar could reduce the crystallization rate and the higher the content of rice husk biochar, the slower the crystallization rate. According to mechanical properties results, the bending strength, tensile strength, and impact strength of BPC were all better than WPC. With the increase of the content of rice husk powder, the bending strength and tensile strength of WPC showed a tendency to rise first and then decrease. The bending strength and tensile strength which could reach 53.7 and 20 MPa of BPC were rising continuously. Although the impact strength of BPC and WPC all decreased with the increase of filler, the impact strength of BPC was still higher than that of WPC, in general. In summary, rice husk biochar is feasible to reinforce HDPE.

## Figures and Tables

**Table 1:** Maximum crystallization peak temperature of different samples at different cooling rate.

Cooling Rate °C/min	Maximum Crystallization Peak Temperature °C					
	HDPE	Biochar—30%	Biochar—40%	Biochar—50%	Biochar—60%	Biochar—70%
5	120.22	119.19	119.44	119.53	119.49	119.39
10	119.33	118.25	118.59	118.55	118.54	118.62
20	118.31	117.37	117.69	117.65	117.78	117.71
30	117.23	116.68	116.32	117.01	117.45	116.98

**Table 2:** Non-isothermal crystallization kinetic parameters of crystallinity at different cooling rates.

Relative Crystallinity	HDPE		BPC (Biochar—30 wt %)		BPC (Biochar—70 wt %)	
	*a*	*F*(*T*)	*a*	*F*(*T*)	*a*	*F*(*T*)
20%	1.60	0.87	1.67	0.92	1.46	1.62
40%	1.61	2.22	1.71	2.58	1.53	5.37
60%	1.65	5.75	2.00	7.65	1.60	11.36
80%	1.70	23.48	2.12	24.90	1.64	29.69

## References

[1] Cicala G., Saccullo G., Blanco I., Samal S., Battiato S., Dattilo S., Saake B. (2017). Polylactide/lignin blends: Effects of processing conditions on structure and thermo-mechanical properties. J. Therm. Anal. Calorim..

[2] Cicala G., Tosto C., Latteri A., La Rosa A.D., Blanco I., Elsabbagh A., Russo P., Ziegmann G. (2017). Green Composites Based on Blends of Polypropylene with Liquid Wood Reinforced with Hemp Fibers: Thermomechanical Properties and the Effect of Recycling Cycles. Materials.

[3] Li S., Li X., Deng Q., Li D. (2015). Three kinds of charcoal powder reinforced ultra-high molecular weight polyethylene composites with excellent mechanical and electrical properties. Mater. Des..

[4] Nguila I.G., Petrissans M., Gerardin P. (2007). Chemical reactivity of heat-treated wood. Wood Sci. Technol..

[5] Hu R., Lim J.-K. (2007). Fabrication and Mechanical Properties of Completely Biodegradable Hemp Fiber Reinforced PLA Composites. J. Compos. Mater..

[6] Jayamani E., Bakri M.K.B. (2015). Dielectric Properties of Lignocellulosic Fibers Reinforced Polymer Composites: Effect of Fiber Loading and Alkaline Treatment. Mater. Today Proc..

[7] Chen J., Wang Y., Gu C., Liu J., Liu Y., Li M., Lu Y. (2013). Enhancement of the Mechanical Properties of Basalt Fiber-Wood-Plastic Composites via Maleic Anhydride Grafted High-Density Polyethylene (MAPE) Addition. Materials.

[8] Schirp A., Stender J. (2010). Properties of extruded wood-plastic composites based on refiner wood fibres (TMP fibres) and hemp fibres. Eur. J. Wood Wood Prod..

[9] Das O., Sarmah A.K., Bhattacharyya D. (2015). A novel approach in organic waste utilization through biochar addition in wood/polypropylene composites. Waste Manag..

[10] Colantoni A., Evic N., Lord R., Retschitzegger S., Proto A.R., Gallucci F., Monarca D. (2016). Characterization of biochars produced from pyrolysis of pelletized agricultural residues. Renew. Sustain. Energy Rev..

[11] Srinivasan P., Sarmah A.K. (2015). Characterisation of agricultural waste-derived biochars and their sorption potential for sulfamethoxazole in pasture soil: A spectroscopic investigation. Sci. Total Environ..

[12] Krishnakumar S., Rajalakshmi A.G., Balaganesh B., Manikandan P., Vinoth C., Rajendran V. (2014). Impact of Biochar on Soil Health. Int. J. Adv. Res..

[13] Sarmah A.K., Srinivasan P., Smernik R.J., Manleyharris M., Antal M.J., Downie A., Zwieten L.V., Krull E., Singh B., Joseph S. (2010). Retention capacity of biochar-amended New Zealand dairy farm soil for an estrogenic steroid hormone and its primary metabolite. Aust. J. Soil Res..

[14] Das O., Bhattacharyya D., Hui D., Lau K.T. (2016). Mechanical and flammability characterisations of biochar/polypropylene biocomposites. Compos. Part B Eng..

[15] Ikram S., Das O., Bhattacharyya D. (2016). A parametric study of mechanical and flammability properties of biochar reinforced polypropylene composites. Compos. Part A Appl. Sci. Manuf..

[16] Zhang Q., Cai H., Ren X., Kong L., Liu J., Jiang X. (2017). The Dynamic Mechanical Analysis of Highly Filled Rice Husk Biochar/High-Density Polyethylene Composites. Polymers.

[17] Khan A., Savi P., Quaranta S., Rovere M., Giorcelli M., Tagliaferro A., Rosso C., Jia C. (2017). Low-Cost Carbon Fillers to Improve Mechanical Properties and Conductivity of Epoxy Composites. Polymers.

[18] Rao A.K., Ahmad S., Savi P., Tulliani J.M., Giorcelli M., Ferro G.A. (2015). Improvement in electromagnetic interference shielding effectiveness of cement composites using carbonaceous nano/micro inerts. Constr. Build. Mater..

[19] Li S., Li D. (2014). Carbon fiber reinforced highly filled charcoal powder/ultra high molecular weight polyethylene composites. Mater. Lett..

[20] Zhang X., Wang H., He L., Lu K., Sarmah A., Li J., Bolan N.S., Pei J., Huang H. (2013). Using biochar for remediation of soils contaminated with heavy metals and organic pollutants. Environ. Sci. Pollut. Res..

[21] Mo Z. (2008). A method for the non-isothermal crystallization kinetics of polymers. Acta Polym. Sin..

[22] Huttepain M., Oberlin A. (1990). Microtexture of nongraphitizing carbons and tem studies of some activated samples. Carbon.

[23] Kyotani T. (2000). Control of pore structure in carbon. Carbon.

[24] Liou T.H. (2004). Preparation and characterization of nano-structured silica from rice husk. Mater. Sci. Eng. A.

[25] You Z., Li D. (2013). The dynamical viscoelasticity and tensile property of new highly filled charcoal powder/ultra-high molecular weight polyethylene composites. Mater. Lett..

[26] Bledzki A.K., Jaszkiewicz A., Scherzer D. (2009). Mechanical properties of PLA composites with man-made cellulose and abaca fibres. Compos. Part A Appl. Sci. Manuf..

[27] Sewda K., Maiti S.N. (2010). Crystallization and melting behavior of HDPE in HDPE/teak wood flour composites and their correlation with mechanical properties. J. Appl. Polym. Sci..

[28] Huang L., Wang H., Wang Q. (2014). The non-isothermal crystallization kinetics analysis of polypropylene based wood plastic composite. Eng. Sci..

[29] Joshi M., Butola B.S. (2004). Studies on nonisothermal crystallization of HDPE/POSS nanocomposites. Polymer.

[30] Ou R., Xie Y., Guo C., Wang Q. (2012). Isothermal crystallization kinetics of Kevlar fiber-reinforced wood flour/high-density polyethylene composites. J. Appl. Polym. Sci..

[31] Shi X., Wang J., Jiang B., Yang Y. (2013). Influence of nanofiller dimensionality on the crystallization behavior of HDPE/carbon nanocomposites. J. Appl. Polym. Sci..

[32] Ahmad I., Chong E.L., Mohd D.H., Abdullah I. (2012). Electron-beam-irradiated rice husk powder as reinforcing filler in natural rubber/high-density polyethylene (NR/HDPE) composites. Compos. Part B Eng..

[33] Kord B. (2011). Nanofiller reinforcement effects on the thermal, dynamic mechanical, and morphological behavior of hdpe/rice husk flour composites. Bioresources.

[34] Lu J.Z., Wu Q., Negulescu I.I. (2010). Wood-fiber/high-density-polyethylene composites: Coupling agent performance. J. Appl. Polym. Sci..

[35] Nurshamila S.B., Ismail H., Othman N. (2012). The effect of Rattan filler loading on properties of Rattan powder filled polypropylene composites. Bioresources.

